# Clinical Characteristics of Autoimmune Hepatitis in a Middle Eastern Population: A Tertiary Care Center Experience

**DOI:** 10.3390/jcm12020629

**Published:** 2023-01-12

**Authors:** Tarek A. Tamimi, Malik Sallam, Deema Rayyan, Randa Farah, Dana Alkhulaifat, Abdallah Al-Ani, Reem Elmusa, Said Sharawi, Omar Tanash, Yaser Rayyan

**Affiliations:** 1 Section of Gastroenterology, Department of Internal Medicine, Jordan University Hospital, Amman 11942, Jordan; 2 Department of Internal Medicine, School of Medicine, University of Jordan, Amman 11942, Jordan; 3 Department of Pathology, Microbiology and Forensic Medicine, School of Medicine, University of Jordan, Amman 11942, Jordan; 4 Department of Clinical Laboratories and Forensic Medicine, Jordan University Hospital, Amman 11942, Jordan; 5 School of Medicine, University of Jordan, Amman 11942, Jordan; 6 Section of Nephrology, Department of Internal Medicine, Jordan University Hospital, Amman 11942, Jordan

**Keywords:** autoantibodies, autoimmune hepatitis, cirrhosis, overlap syndrome, primary biliary cholangitis, clinical hepatology, diagnosis

## Abstract

Autoimmune hepatitis (AIH) is an immune-mediated inflammatory liver disease of uncertain cause, and its manifestations appear to vary by race and ethnicity. The literature on AIH in the Middle East, including Jordan, is scarce; therefore, this study aimed to determine the clinical characteristics of AIH in an understudied population. This retrospective chart review study was conducted on AIH patients who presented to Jordan University Hospital over a seven-year period (2014–2020). Retrieved data included sociodemographics, liver function tests, autoimmune serologic markers, viral hepatitis serology, findings on liver biopsies, treatment regimens, post-therapy outcomes and treatment-related complications. The total number of AIH patients included in the study was 30, divided as follows: type 1 AIH (*n* = 17, 56.7%), type 2 AIH (*n* = 2, 6.7%), seronegative AIH (*n* = 9, 30.0%), and two patients who had AIH-primary biliary cirrhosis overlap syndrome (6.7%). The mean age at diagnosis was 44 years (standard deviation: 17 years), with a female predominance (*n* = 25, 83.3%). Acute presentation was seen among 18 patients (60.0%). Mild to moderate fibrosis (F1 and F2 on METAVIR scoring system) without cirrhosis was observed among patients who underwent liver biopsies (10/19, 52.6%). The majority of patients (73.3%) were initially treated with prednisone, with azathioprine combination in 16.7% of the patients. At 6 months post initial treatment, twenty patients (66.7%) achieved biochemical remission, four patients had incomplete response, two patients failed to improve (one died during the induction of remission period due to AIH-related complications), and four patients were lost to follow-up. This study provided an updated overview of AIH in Jordan. The results showed typical female predominance, and interestingly high rates of acute presentation and seronegative disease. Future longitudinal studies are recommended to address the nature and long-term prognosis of AIH in Jordan.

## 1. Introduction

Autoimmune hepatitis (AIH) is an immune-mediated inflammatory liver disease of uncertain etiology [1,2,3,4]. It is primarily characterized by necro-inflammatory changes in the liver, accompanied by circulating autoantibodies with hypergammaglobulinemia [5,6]. The spectrum of AIH clinical presentation ranges from isolated asymptomatic elevation of liver enzymes, acute hepatitis, chronic disease that can lead to cirrhosis, and hepatocellular carcinoma (HCC) to the dramatic acute liver failure presentation with coagulopathy and encephalopathy [1,2,7].

The pathogenesis of AIH involves loss of tolerance to hepatic antigens, likely triggered by a complex interaction between environmental factors, immunoregulatory pathways and genetic predisposition [8,9]. Such roots are best exemplified by association of AIH with different autoimmune disorders such as type 1 diabetes mellitus, autoimmune thyroiditis, colitis, celiac disease, and systemic lupus erythematosus [10,11].

The occurrence of AIH is evident in children and adults, with a worldwide incidence ranging from 0.7 to 2.0 per 100,000 individuals per year, while its prevalence ranges from 4 to 25 per 100,000 individuals [12,13]. The disease is characterized by a significant predilection for females with 75–80% of patients being females, yet AIH can manifest itself in males, as well as in all ethnic and age groups [1,14].

The classification of AIH relies on the autoantibody profile of patients; nevertheless, histopathologic examination of liver biopsies is essential to confirm the AIH diagnosis [15,16]. Type 1 AIH is the most prevalent comprising about 80% of all AIH cases, with the detection of antinuclear antibodies (ANAs) and the frequent presence of anti-smooth muscle antibody (SMA). Type 2 autoimmune hepatitis is characterized by the detection of liver–kidney microsomal antibodies (LKM) or anti–liver cytosol type 1 antibodies (LC) at a lower frequency. The presence of antimitochondrial antibody (AMA) is the hallmark of primary biliary cholangitis (PBC) which may signify an underlying autoimmune-PBC overlap syndrome. Other less frequently detected autoantibodies include anti-soluble liver antigen (SLA) found in all types of AIH, and anti-perinuclear neutrophil cytoplasm antibodies (pANCA) in primary sclerosing cholangitis (PSC) and type 1 AIH [17,18,19,20,21]. In 10–14% of cases, the patients may present with an atypical AIH clinical picture dominated by PSC, or PBC known as overlap syndrome, or a seronegative picture devoid of circulating antibodies (seronegative AIH, cryptogenic AIH), at least in the initial phases of the disease [22,23].

The treatment regimen is similar for all types of AIH, yet each has a different prognosis in terms of severity or long-term outcome [18,24,25]. Despite AIH’s ambiguous presentation and distorted histological features, it responds favorably to a combination therapy of glucocorticoids and immunosuppressive agents [26]. Nonetheless, AIH is overlooked and underdiagnosed throughout Asia and the Middle East due to the high prevalence of hepatitis B and C within the region [27,28,29]. This led to an overall scarcity of reports addressing the nature of the disease in the region. Therefore, the aim of this study was to determine the characteristics of AIH patients among Jordanian population in terms of clinical features, biochemical profile, and response to treatment. Moreover, the study addresses the seronegative presentations of AIH, acute presentation of AIH, and compares them to relevant literature.

## 2. Materials and Methods

### 2.1. Study Design

We performed a retrospective analysis of all patients diagnosed with AIH who were followed at the Gastroenterology and Hepatology clinics at Jordan University Hospital (JUH), Amman, Jordan, from May 2014 to March 2020. Patients’ demographic data, clinical history at presentation, laboratory and serologic testing results at diagnosis were extracted as well. Liver biopsy results were collected when available. Moreover, patients’ treatment regimens, response to treatment, and post-treatment complications were obtained.

The inclusion criteria were patients diagnosed with probable or definite AIH, based on the simplified criteria for the diagnosis of AIH, who were followed at the Gastroenterology and Hepatology clinics at JUH, Amman, Jordan, from May 2014 to March 2020.

The exclusion criteria were as follows: (1) inconclusive diagnosis of AIH; (2) patients with incomplete medical records; and (3) patients presenting with co-existing liver diseases such as viral hepatitis, non-alcoholic fatty liver disease, or alcohol-associated liver disease with alcohol consumption >25 g/day. The patients were also assessed for the presence of concomitant autoimmune diseases.

### 2.2. Clinical Chemistry Laboratory Profile

The clinical chemistry laboratory testing at time of diagnosis was performed using Roche module Cobas 6000 (C-501, Roche Diagnostics International AG, Rotkreuz ZG, Switzerland) and consisted of the following: liver enzymes (alanine aminotransferase [ALT; normal, 7–55 U/L], aspartate amino transferase [AST; normal, 8–48 U/L], alkaline phosphatase [ALP; normal, 40–129 U/L], gamma-glutamyl transferase [GGT; normal, 8–61 U/L]), albumin [Normal, 3.5–5.0 g/dL], total and direct bilirubin [normal, 0.1–1.2 and 0–0.035 mg/dL, respectively], prothrombin time [PT; normal, 9.4–12.5 s], prothromboplastin time [PTT; normal, 32–46 s], and International Normalized Ratio [INR; normal, × <1.1]. Kidney function tests at the time of diagnosis were also recorded (serum creatinine [SCr; normal, 0.6–1.2 and 0.5–1.1 mg/dL, for males and females, respectively], blood urea nitrogen [BUN; normal, 9–20 mg/dL], and glomerular filtration rate [GFR; normal, 90–120 mL/min/1.73 m^2^]).

### 2.3. Assessment of the Serologic Profile and Histopathologic Evaluation of Liver Biopsies

The patients’ serologic profiles included testing for ANA, SMA, LKM, and AMA by indirect immunofluorescence testing using HEp2 cells (Bio-Rad, 1000 Alfred Nobel Drive, Hercules, CA 94547, USA) and rodent substrate including kidney, liver, and stomach cells (ZEUS IFA Autoantibody Screen, ZEUS Scientific, 199 & 200 Evans Way, Branchburg, NJ 08876, USA). The determination of the total immunoglobulin G (IgG) titer (reference range for individuals: 7–16 g/L) was performed using COBAS INTEGRA 400 plus analyzer—(Roche Diagnostics International AG, Rotkreuz ZG, Switzerland). The determination of pANCA was based on indirect enzyme-linked immunosorbent assay (ELISA, MPO-ANCA Generic Assays, GA Generic Assays GmbH, Blankenfelde-Mahlow, Germany). Viral hepatitis serology (HAV IgM, HBsAg, HBeAg, and HCV IgG) was based on chemiluminescent immunoassay using ARCHITECT Clinical Chemistry Analyzer (Abbott Laboratories, 100 Abbott Park Rd, Abbott Park, IL 60064, USA). Other viral hepatitis tested were Herpes simplex virus (HSV), Cytomegalovirus (CMV), and Epstein-Barr virus (EBV) as per the discretion of treating physician, which were conducted using Uniplex real-time polymerase chain reactions. Liver biopsies were evaluated by the histopathologists at JUH for the presence and degree of interface hepatitis, bridging necrosis, lymphoplasmacytic infiltrate, presence of rosette pattern, fibrosis score, and overall grade of inflammation when available. The METAVIR scoring system was used to grade inflammation and stage fibrosis [30].

### 2.4. The Criteria for the Diagnosis of AIH and the Evaluation of Response to Treatment

The diagnosis of AIH is based on the simplified criteria for the diagnosis of AIH established by of the International Autoimmune Hepatitis Group (IAIHG), through a constellation of findings consisting of the following: (a) positive ANA or SMA [titers ≥ 1:40] (1 point), positive ANA or SMA [titers ≥ 1:80] or LKM [titers ≥ 1:40] (2 points); (b) IgG titers over the upper limit of normal (1 point), or over 10% above the upper limit of normal (2 points); (c) liver histology compatible with AIH (1 point), or typical of AIH (2 points); (d) absence of viral hepatitis (2 points). Probable and definite AIH diagnoses are defined as a sum score of ≥6 and ≥7, respectively [31].

The patients were further classified as having type 1 AIH if positivity for ANA and/or SMA was detected; type 2 AIH if positivity for LKM was found; and seronegative AIH if the patients were negative for ANA, SMA, and LKM autoantibodies, in spite of the presence of other characteristic features of AIH. The “Paris criteria” were used to identify patients with AIH-PBC overlap syndrome, as reported by Chazouilleres et al. [32]. Two of the following three criteria for each of PBC and AIH should be met: (a) PBC criteria: (1) serum ALP ≥ 2-fold the upper limit of normal (ULN) range or serum GGT level ≥ 5-fold ULN, (2) the presence of AMA, and (3) florid bile duct lesions on histological examination; (b) Criteria for AIH in the setting of PBC: (1) serum ALT level ≥ 5-fold ULN, (2) serum IgG level ≥ 2-fold ULN or the presence of SMA, and (3) a liver biopsy showing moderate or severe periportal or periseptal lymphocytic piecemeal necrosis [23].

Treatment evaluation included data on treatment regimens, duration, and related complications. Such data were obtained for both induction and maintenance therapy. Response to treatment was assessed through patients’ biochemical responses at 6-month, 1–year, and 2-year intervals. Biochemical remission is defined as normalization of serum AST, ALT, and IgG levels 24 months post therapy. Incomplete response was defined as improvement in laboratory and histological findings that are insufficient to satisfy criteria for remission. Treatment failure was defined as worsening of laboratory or histological findings, despite compliance with standard therapy. Other treatment endpoints were defined similar to the American Association for the Study of Liver Disease’s (AASLD) guidelines [3,33].

### 2.5. Statistical Analysis

Statistical analysis of all data was conducted using the statistical package for social sciences (SPSS) 22.0 (SPSS Inc., Chicago, IL, USA). Continuous data were reported as means and standard deviations, while categorical data were reported as frequencies [*n* (%)]. Associations between categorical variables were assessed using the chi-squared test (χ^2^). Two-tailed Mann–Whitney *U* test (M–W), and Kruskal–Wallis test (K–W) were used to investigate possible associations between scale variables and categorical variables. *p* values < 0.050 were considered statistically significant.

## 3. Results

### 3.1. Clinical Characteristics of the Study Population

**Figure 1:**
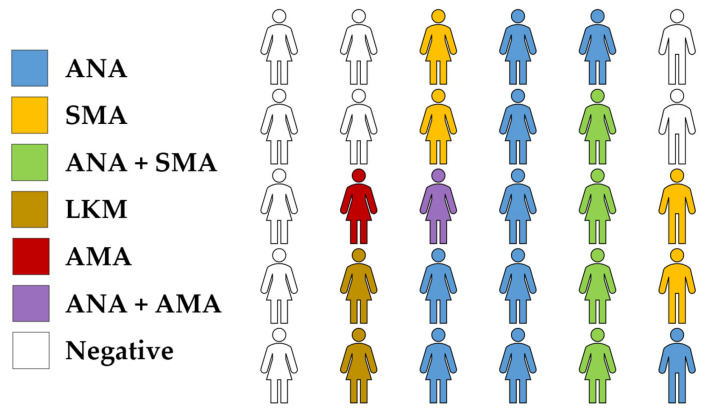
Characteristics of the study participants based on sex and serologic profile. ANA: Antinuclear antibodies; SMA: anti-smooth muscle antibody; LKMs: liver–kidney microsomal antibodies; AMA: antimitochondrial antibody.

The total number of participants that was included in final analysis was 30, with female predominance (*n* = 25, 83.3%, Figure 1).

The mean age of participants was 44 ± 17 years, while the mean BMI was 25.3 ± 6.3 kg/m^2^. Eighteen patients (60.0%) had acute presentation with manifestations varying between signs of acute hepatitis and acute liver failure. On the other hand, 12 patients (40.0%) had chronic hepatitis manifesting either asymptomatically, or with mild constitutional symptoms. Type 1 AIH was the most frequent type (*n* = 17, 56.7%), while two patients had type 2 AIH (6.7%), two patients had AIH-PBC overlap syndrome (6.7%), and nine patients had seronegative AIH (30.0%, Figure 1). Associated autoimmune disorders were present in six patients, as follows: one patient had Hashimoto’s thyroiditis, one had vitiligo and psoriasis, one had rheumatoid arthritis, one had celiac disease, one had ulcerative colitis, and one patient with overlap syndrome had Sjögren’s syndrome. Using the simplified criteria for the diagnosis of AIH, of the 19 patients who underwent liver biopsies, 8 of them had definite AIH (≥7 points), while 11 patients had probable AIH (6 points). Regarding the 11 patients who did not undergo a liver biopsy, all of them had probable AIH with a score of 6 points which is the maximum score that can be achieved if no biopsy is performed.

### 3.2. Baseline Laboratory Parameters at Time of Diagnosis

The mean levels of the various laboratory panels at time of diagnosis stratified by AIH types are summarized in (Table 1). No statistically significant differences were found for all of the laboratory parameters upon comparing the four different AIH types. Viral hepatitis serology was negative in all patients for hepatitis A, B, and C antibodies. The autoantibody profile for the patients showed positive ANA in 46.7% of the patients (*n* = 14), positive SMA in 8 out of 28 (28.6%) patients, positive LKM antibody in 2 out of 23 (8.7%) patients, and positive AMA in 2 out of 27 (7.4%) patients.

### 3.3. Histopathologic Evaluation of Liver Biopsies

Baseline liver biopsies were obtained from 19 patients (63.3%). All biopsies’ results were consistent with AIH. Liver fibrosis, based on the METAVIR scoring system, ranged from no fibrosis (F0, *n* = 2), mild fibrosis (F1, *n* = 7), moderate fibrosis (F2, *n* = 6), to severe fibrosis (F3, *n* = 4); none of the examined biopsies showed F4 fibrosis/cirrhosis (Table 2). The presence of lymphoplasmacytic infiltrate was seen among all cases of type 1 AIH, type 2 AIH, and seronegative AIH, as opposed to 50% in AIH-PBC overlap syndrome (*p* = 0.030, χ^2^ test). Higher grades of inflammation (A2 and A3) were found among type 1 AIH patients (100%), seronegative AIH (83.3%), AIH-PBC overlap syndrome (50.0%) compared to its total absence (A2 and A3 grades of inflammation) in type 2 AIH (*p* = 0.046, χ^2^ test).

### 3.4. Treatment Regimens, Response, and Side Effects

All patients received induction treatment by prednisone with or without azathioprine. Maintenance therapy involved using both prednisone and azathioprine (*n* = 20, 66.7%), or prednisone only (*n* = 5, 16.7%). Mycophenolic acid (*n* = 3, 10%) and budesonide (*n* = 1, 3.3%) were used for patients with azathioprine intolerance or standard therapy failure. One patient died during the induction phase due to acute liver failure complicated by multiorgan failure. At the 6-month follow-up mark, twenty patients (66.7%) achieved biochemical remission, four patients (13.3%) had incomplete response, two patients failed to improve (one died during the induction of remission period), and four patients were lost to follow up. Of those who achieved biochemical remission at 6 months, eleven patients had type 1 AIH, eight had seronegative AIH, and one had type 2 AIH. Additionally, eleven patients (55%) out of the biochemical responders had an acute presentation. Concomitant induction with azathioprine, in addition to prednisone, was delayed due to the high percentage of acute presentation and compromised liver function tests.

At the 1-year follow-up mark, 10 patients had been lost to follow-up. Out of the remaining 19 patients undergoing follow-up at our center at that time, 15 patients (78.9%) had biochemical remission, and 4 patients experienced treatment failure. Of those who achieved biochemical remission at 1 year, 12 patients had type 1 AIH, and 3 patients had seronegative AIH. Patients’ characteristics based on their response to treatment are delineated in Table 3.

**Figure 2:**
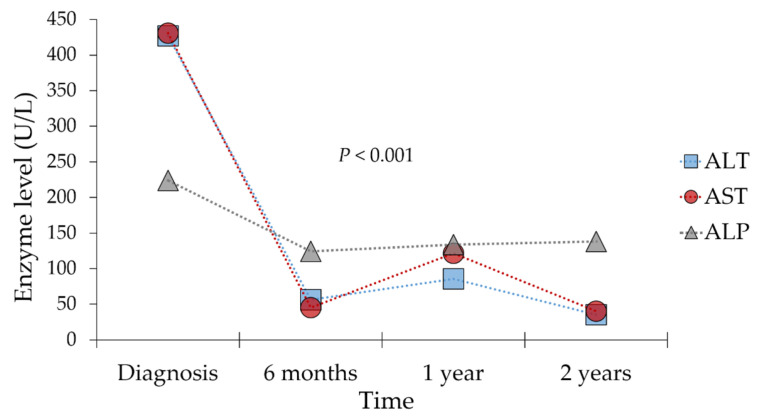
Response to treatment among patients at different time intervals as evidenced by liver function tests. ALT: Alanine aminotransferase in U/L; AST: Aspartate aminotransferase in U/L; ALP: Alkaline phosphatase in U/L. *p* values were calculated using the Kruskal–Wallis test.

The progression of the patients’ mean liver enzyme levels are presented in Figure 2, with significant reduction of ALT, AST, and ALP levels from diagnosis over a period of two years. The mean ALT level decreased from a mean value of 427.1 U/L at time of diagnosis to 35.3 U/L two years later (*p* < 0.001, K–W). For AST, the decline was from a mean value of 430.8 U/L at time of diagnosis to a mean level of 40.3 U/L after two years (*p* < 0.001, K–W). For ALP, the decline was from a mean level of 223.7 U/L at time of diagnosis to a mean value of 138.0 U/L following two years (*p* < 0.001, K–W, Figure 2). Side effects of treatment were noted in 12 patients (40.0%). The most common side effect was hypertension (23.3%), followed by weight gain (13.3%), osteoporosis (10.0%), and glucose intolerance (10.0%) among the entire 30-patient cohort. Follow up durations for this study ranged from 2 months to 13 years, with mean and median follow up durations of 4.4 and 2.7 years, respectively.

## 4. Discussion

In the current study, we characterized the serologic, biochemical, and histopathologic profile of the patients with AIH for the first time in Jordan. Taking into consideration the rarity of the disease worldwide with a prevalence ranging between 4 and 25 per 100,000 individuals, the current study involved about 1–10% of the estimated total number of AIH cases in the country. Thus, the findings of the study can be representative of a considerable number of AIH cases in Jordan, and can pave the way for more comprehensive work on AIH in the region due to limited research addressing this objective in the Middle East [34].

Despite the lack of accurate estimates on the prevalence of AIH in Jordan, the previous evidence of an association between more northern latitude and autoimmune disease in general and AIH in particular may hint to the very low prevalence of AIH in Jordan. Thus, the previous assumption can support the representativeness of our sample despite the small number of patients included in the study. The likely explanation of the link between the northern latitude and autoimmune disease is related to vitamin D deficiency and insufficiency with subsequent higher risk of autoimmunity [35,36,37]. The very low prevalence of AIH in the Middle East was seen previously in a retrospective study from Jeddah, Saudi Arabia, where only 41 patients were diagnosed with AIH over a period of 15 years [29].

The major findings of this study can be summarized as follows: our study cohort showed the typical predilection of AIH occurrence among females, who represented 83.3% of the patients. Previous observations of female predominance in AIH can be attributed to immunogenetic factors and sex hormones, despite the enigmatic nature of such a pattern of sex difference in autoimmunity in general [14,38]. The pattern of female predominance was also seen among AIH patients in previous and recent studies from the US and UK (77%), Italy (88%), Japan (79%), Saudi Arabia (76%), Brazil (75%), and Egypt (60% in a small sample of children) [29,35,39,40,41,42,43]. Additionally, and consistent with the previous literature, a large variability in terms of age at time of diagnosis was observed in this cohort, and age ranged from as young as 16 years up to 82 years. In general, age at the time of AIH diagnosis is associated with distinctive presentation (with less frequency of acute manifestations among the elderly), but not response to treatment [44,45]. The mean age at time of AIH diagnosis in this study was 44 years which is slightly older compared to another study from Saudi Arabia (32 years), while a recent Japanese cohort reported a much older average age of 65 years among AIH patients in Ueda city [29,42].

Regarding the clinical picture of AIH in this study, 60% of the patients had acute presentation which is similar to previous studies from Sweden and Canada, while the rate of acute presentation was much lower (13%) in an earlier study from India [46,47,48]. The majority of acute AIH cases in this study manifested as emergency visits provoked through acute symptomatic flares and elevated markers of coagulation [49]. This can be explained by the lack of regular screening visits and delayed medical evaluation for most patients due to the vague nature of early clinical presentations. The wide spectrum of AIH clinical presentation in different regions can be linked to gene disparity and antigen trigger variability [50,51,52].

Another finding of this study is the predominance of type 1 AIH among other types, which was noted in various studies across the globe [29,49,53]. In general, AIH is categorized into two types based on autoantibody profile. The different variants of autoantibody profiles are a phenotypic feature that aids in diagnosis. The disparity of serological manifestations does not encompass any pathological significance and is common between age groups of various countries [54]. In this study, out of 30 patients that had undergone complete serological testing, 57% had type 1 AIH, 2 patients had type 2 AIH, and 30% were labeled as having seronegative AIH. The two patients characterized with AIH type 2 were aged 21 and 43 years old. This finding is interesting as AIH type 2, characterized by the presence of LKM antibodies, is typically found in pediatric populations and is only present in about 3% of the US population with AIH, indicating a rare occurrence in adults [55,56]. Variable results of AIH type 2 prevalence among all AIH cases were reported in different Middle Eastern studies, ranging from total absence to 14% [29,49,57,58].

Our sample shows a moderately high rate of seronegative AIH among participants (30.0%). This rate is higher than any documented in the literature [49,57,59,60]. Such discrepancy might reflect regional differences in terms of antigen exposure which are predetermined by pre-set genetic predisposition. It should be noted that patients with negative serologic markers exhibit similar characteristics and response to treatment to that of typical AIH types [61,62]. Similarly, in this study, there were no statistically significant differences between seronegative AIH and other forms of AIH in terms of laboratory results at presentation, histological findings, and response to treatment.

The treatment of AIH is targeted to alleviate inflammation and fibrosis, and prevent the disease progression to cirrhosis [63]. Monotherapy with prednisolone or combination therapy with prednisolone/azathioprine are common regimens initiated for AIH management [64]. Our study shows that 73% of patients were induced by prednisolone alone. Later on, 66.7% of patients were maintained on a combination of steroids and azathioprine. Of the patients followed up till the 6-month mark, 66.7% achieved biochemical remission, and 20% experienced either incomplete response or treatment failure. Our response rate is higher than that in Saudi Arabia (54.8%) and similar to the global remission rate of 65% at 18 months [29,59]. Treatment failure can be attributed to post-therapy complications such as intolerance, hypertension, diabetes mellitus, osteoporosis, and weight gain, all of which might necessitate either dose reductions or contribute to drug withdrawal or non-compliance [59].

The current study had several limitations that should be considered in the interpretation of results, including the retrospective design, with inherent caveats in the investigation of rare diseases [65]. In addition, the data used to conduct the current study relied on medical records; therefore, some data may be prone to having a poor quality, with a few missing variables. Moreover, a loss to follow-up was encountered in our study. Furthermore, the small sample size should be considered in any future research addressing AIH in the country, with possible utility of multi-center study design. Finally, the current study was based on a tertiary care teaching hospital in Amman, with visitors mainly from the central region of Jordan, implying that the sample might not be representative of the whole country.

## 5. Conclusions

In conclusion, this study illustrates the clinical and immunological characteristics of AIH in Jordan. Besides female predominance, high rates of acute presentation, seronegative AIH, and mild to moderate degrees of fibrosis without cirrhosis were observed, implying a possible regional difference related to serologic profile of AIH patients in Jordan. Therefore, we suggest creating a Jordanian national database for future evaluation of this rare disease, building on the results of the current pilot study.

## Figures and Tables

**Table 1:** Characteristics of the various laboratory panels at time of diagnosis stratified by autoimmune hepatitis (AIH) types in the study cohort.

Variable	Type 1 AIH	Type 2 AIH	AIH-PBC ^12^ Overlap Syndrome	Seronegative AIH	*p* Value ^13^
Age	45, 43 (32–61)	32, 32 (21–43)	45, 45 (34–55)	43, 39 (35–53)	0.816
BMI ^1^	25.2, 25.8 (21.4–27.7)	25.8, 25.8 (22.9–28.7)	24.8, 24.8 (18.4–31.2)	25.6, 23.3 (21–30.2)	0.998
ALT ^2^	390.9, 298 (61–667)	1044.4, 1044.4 (893.6–1195.2)	78.6, 78.6 (65–92.2)	435.7, 195.9 (63–288)	0.245
AST ^3^	396.5, 337.2 (50–620)	996.4, 996.4 (695.2–1297.5)	52.5, 52.5 (30–74.9)	454.1, 203.4 (46–357)	0.209
GGT ^4^	259, 197.3 (100–269)	131, 131 (106–156)	618, 618 (371–865)	561.1, 128 (71–234)	0.351
ALP ^5^	233.2, 190 (124–294)	205, 205 (139–271)	247, 247 (117–377)	204.8, 214 (168–232)	0.979
Total Bilirubin (mg/dL)	5.761, 5.458 (1.09–8.2)	8.626, 8.626 (2.975–14.276)	0.53, 0.53 (0.5–0.56)	11.274, 1.9 (1.45–20.98)	0.213
Direct Bilirubin (mg/dL)	4.601, 3.92 (0.44–7.19)	7.163, 7.163 (1.718–12.607)	0.205, 0.205 (0.1–0.31)	9.452, 1.6 (0.62–18.7)	0.271
PT ^6^	17.1, 16.3 (14.6–18.2)	17.8, 17.8 (16.6–19)	12.6, 12.6 (12.1–13)	15.8, 14.3 (13.6–16.6)	0.083
PTT ^7^	34.1, 33.1 (30.1–36.4)	35.2, 35.2 (33.1–37.3)	29, 29 (28.4–29.5)	34.3, 33.6 (30.8–38.5)	0.446
INR ^8^	1.35, 1.32 (1.12–1.41)	1.42, 1.42 (1.25–1.59)	0.93, 0.93 (0.89–0.97)	1.2, 1.13 (1.03–1.3)	0.080
Albumin (g/dL)	3.56, 3.8 (3.29–3.9)	3.25, 3.25 (2.52–3.98)	4.4, 4.4 (4.3–4.5)	3.76, 3.8 (3.4–4.3)	0.155
Creatinine (mg/dL)	0.42, 0.42 (0.3–0.49)	0.62, 0.62 (0.5–0.74)	0.63, 0.63 (0.58–0.68)	0.5, 0.4 (0.14–0.46)	0.135
BUN ^9^	23.6, 21 (17–26)	24, 24 (15.8–32.3)	28.6, 28.6 (28.1–29)	24.2, 20 (13–25)	0.671
GFR ^10^	135, 130 (119–155)	118.5, 118.5 (99–138)	109, 109 (104–114)	142.9, 125.5 (114–160.5)	0.426
IgG ^11^	20.89, 19.56 (14.2–26.67)	22.37, 22.37 (17.72–27.02)	13.52, 13.52 (8.97–18.08)	15.19, 15.84 (13.01–19.12)	0.253

^1^ BMI: Body mass index in kg/m^2^; ^2^ ALT: Alanine aminotransferase in U/L; ^3^ AST: Aspartate aminotransferase in U/L; ^4^ GGT: Gamma-glutamyl transferase in U/L; ^5^ ALP: Alkaline phosphatase in U/L; ^6^ PT: Prothrombin time in seconds; ^7^ PTT: Partial thromboplastin time in seconds; ^8^ INR: International normalized ratio; ^9^ BUN: Blood urea nitrogen in mg/dL; ^10^ GFR: Glomerular filtration rate in mL/min/1.73 m^2^; ^11^ IgG: Immunoglobulin G titer in g/L; ^12^ PBC: Primary biliary cholangitis; ^13^ *p* value: Calculated using the Kruskal–Wallis test. Data were presented as mean, median, and range.

**Table 2:** Histopathologic characteristics of autoimmune hepatitis patients included in the study.

Feature	Degree/Class	Number (%)
Presence of interface hepatitis		18 (94.7%)
Degree of interface hepatitis	Mild	8 (42.1%)
	Moderate	9 (47.4%)
	Severe	1 (5.3%)
Presence of bridging necrosis		9 (52.6%)
Presence of lymphoplasmacytic infiltrate		18 (94.7%)
Degree of lymphoplasmacytic infiltrate	Mild	4 (21.0%)
	Moderate	12 (63.2%)
	Severe	2 (10.5%)
Presence of rosette pattern		3 (15.8%)
Fibrosis score	F0	2 (10.5%)
	F1	7 (36.8%)
	F2	6 (31.6%)
	F3	4 (21.0%)
	F4	0
Grade of inflammation	A0	1 (5.3%)
	A1	3 (15.8%)
	A2	11 (57.9%)
	A3	4 (21.0%)

**Table 3:** Characteristics of the various laboratory panels at time of diagnosis stratified by autoimmune hepatitis (AIH) types in the study cohort.

Outcome	Biochemical Remission at Six Months	Incomplete Response at Six Months	Failed Response at Six Months	Biochemical Remission at One Year	Incomplete Response at One Year	Failed Response at One Year
Total	20 (100%)	4 (100%)	2 (100%)	15 (100%)	-	4 (100%)
Type 1 AIH ^1^	11 (55%)	3 (75%)	2 (100%)	12 (80%)	-	1 (25%)
Type 2 AIH	1 (5%)	1 (25%)	-	0 (%)	-	-
Seronegative AIH	8 (40%)	-	-	3 (20%)	-	3 (75%)
Acute presentation	11 (55%)	4 (100%)	2 (100%)	8 (53%)	-	4 (100%)
Azathioprine at induction	3 (15%)	2 (50%)	0 (0%)	4 (27%)	-	0 (0%)
Mean ALT at diagnosis ^2^	358.6 ± 389.6	594.5 ± 466.0	485.6 ± 401.1	291.3 ± 362.4	-	424.0 ± 451.9
Mean AST at diagnosis ^3^	378.3 ± 440.5	470.9 ± 285.5	387.5 ± 266.6	265.7 ± 308.6	-	470.9 ± 580.7
Total bilirubin at diagnosis ^4^	6.9 ± 7.7	4.5 ± 3.5	4.2 ± 3.5	4.1 ± 4.3	-	5.9 ± 7.1
Direct bilirubin at diagnosis	5.6 ± 6.7	3.5 ± 3.1	3.4 ± 3.1	3.13 ± 4.0	-	5.1 ± 6.3

^1^ AIH: Autoimmune hepatitis; ^2^ ALT: Alanine aminotransferase in U/L; ^3^ AST: Aspartate aminotransferase in U/L; ^4^ Bilirubin: Measured in mg/dL.

## Data Availability

The data presented in this study are available on request from the senior corresponding author (Y.R.).
